# P-177. Analysis of Leishmaniasis and Chagas Disease from 1994-2024 in the Military Medical Database

**DOI:** 10.1093/ofid/ofaf695.401

**Published:** 2026-01-11

**Authors:** Elena Crouch, Lauren Sweet, John Kiley

**Affiliations:** Brooke Army Medical Center, San Antonio, TX; Brooke Army Medical Center, San Antonio, TX; BAMC, San Antonio, Texas

## Abstract

**Background:**

The burden of emerging tropical arthropod-borne diseases in the United States (US) is increasing, but its true prevalence remains undefined. Chagas disease (CD) and Leishmaniasis (LM) are two emerging arthropod-borne diseases which are not yet considered endemic to the US, but whose vectors exist domestically. The US military operates in environments which place them at increased risk of these diseases, both at home and abroad. Using the Defense Medical Surveillance System (DMSS), we evaluated the prevalence and demographics of LM and CD diagnosed within the military system.

**Figure:**
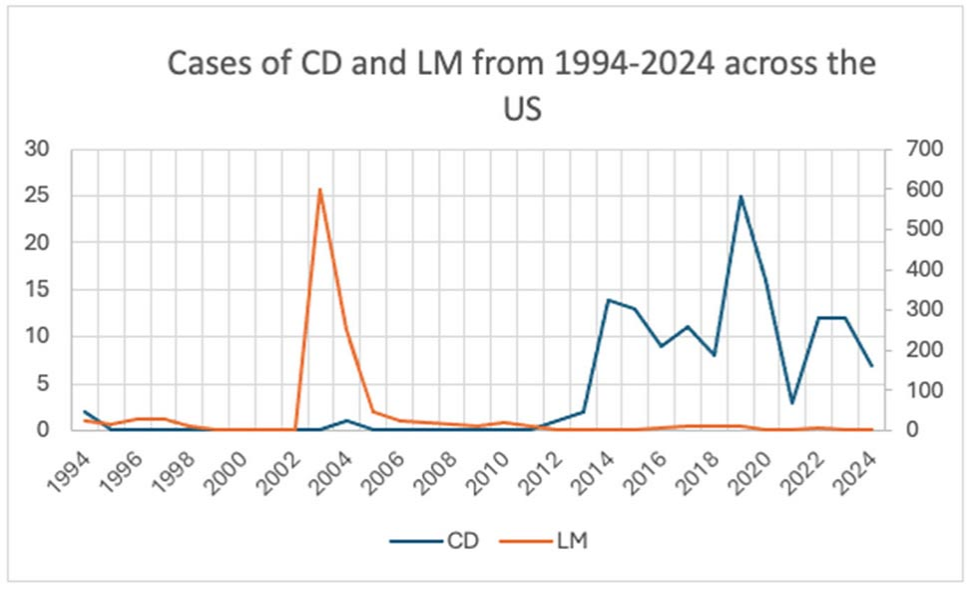
Cases of CD and LM from 1994-2024 Across the US CD= Chagas Disease, LM= Leishmaniasis

**Figure:**
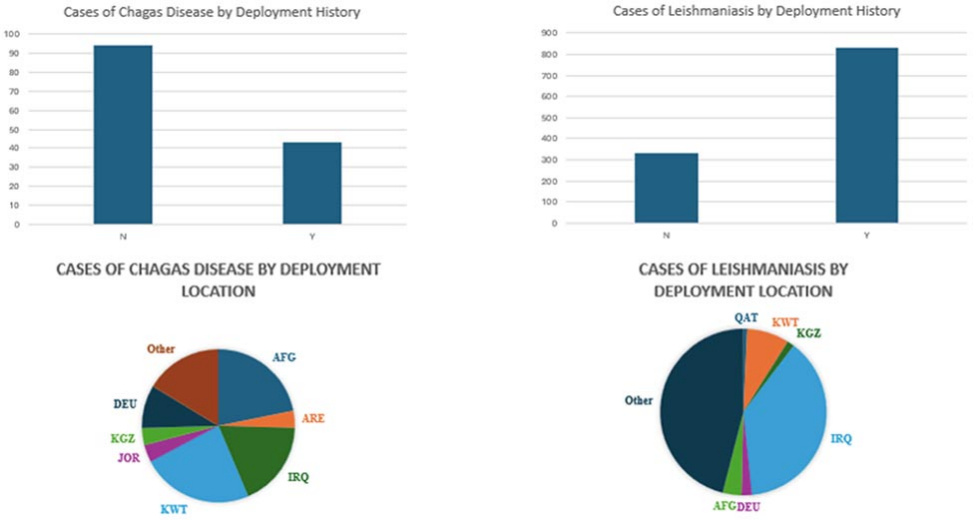
Cases by Deployment and Deployment Location Pie chart abbreviations: DEU= Germany, JOR= Jordan, KWT= Kuwait, AFG= Afghanistan, IRQ= Iraq, QAT= Qatar, ARE=United Arab Emirates, KGZ= Kyrgyzstan. Countries in the "Other" category comprised less than 2% of total cases for CD and LM.

**Methods:**

A retrospective descriptive study of epidemiological data in the DMSS was performed. The DMSS was queried for all confirmed and suspected cases of LM and CD between 1994 and 2024. Confirmed (reportable medical event or positive lab result) and suspected (1 inpatient or 2 outpatient visits within 60 days with a disease-associated ICD-code) cases were included. Confirmed and suspected case definitions were defined a priori by the DMSS.

**Figure:**
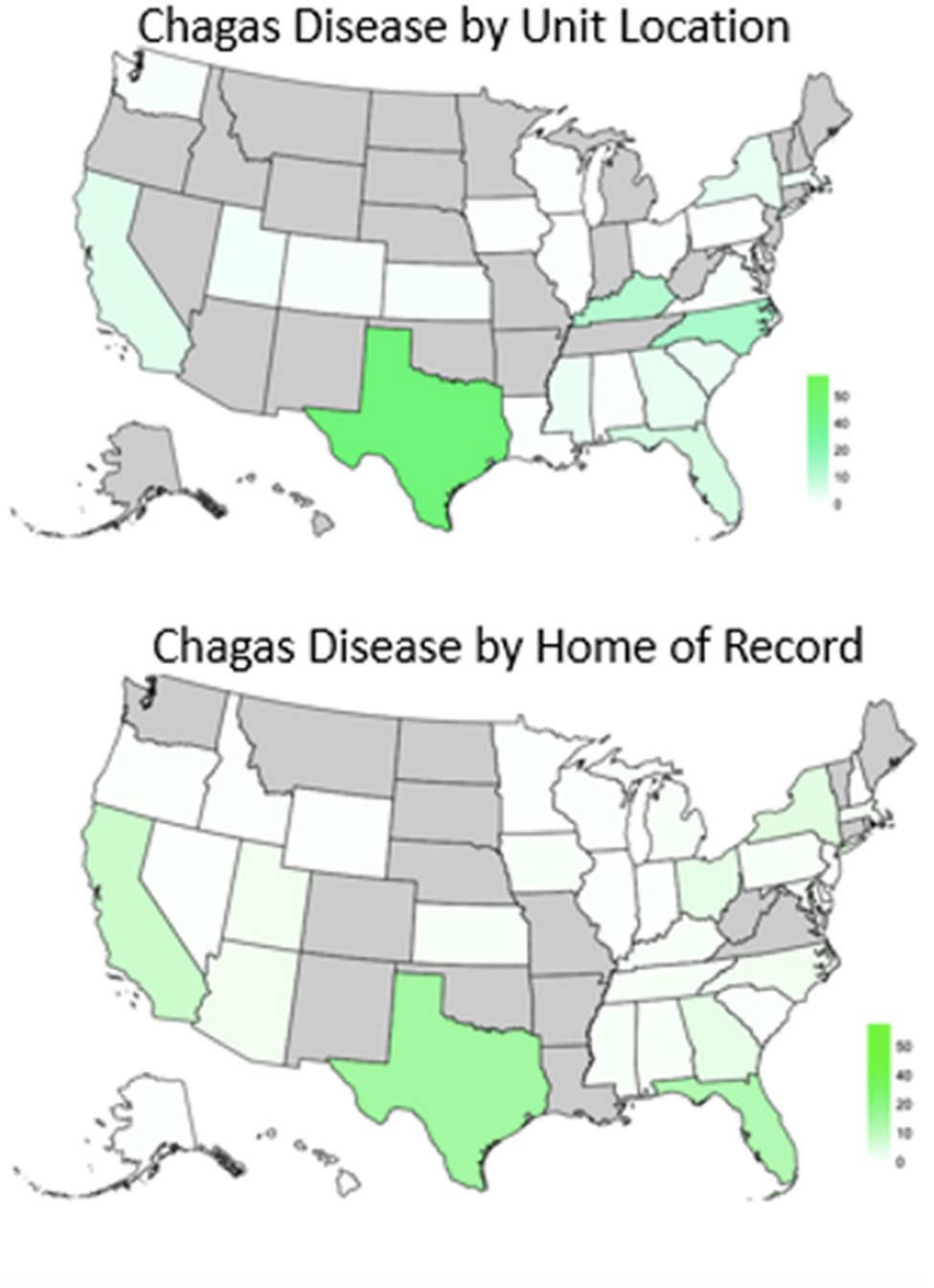
Chagas Disease Distribution Cases by unit location indicates where the service member was stationed at the time of sample collection. Cases by home of record indicates where that service member's home of record was.

**Results:**

Overall, 1166 cases of LM and 137 cases of CD were identified from 1994-2024. There was a decline in LM cases and an increase in CD cases from 1994-2024 (Fig 1). LM was more prevalent in uniformed service members (USM) who had completed a deployment (833 vs 333), while CD was more prevalent in those who had not (43 vs 94) (Fig 2). For LM, there were 992 cases (85% of total) between 2003-2011 and 57 cases (4.8%) from 2012-2024. The deployment locations with the most cases of LM were Iraq (38%) and Kuwait (8.2%). The states with the highest prevalence of CD by home of record were Texas (13.1%), Florida (11%), and California (7.3%). The states in which USM were stationed at time of testing with the highest prevalence for CD were Texas (29.2%), North Carolina (11.7%) and Kentucky (10.2%) (Fig 3).

**Conclusion:**

LM case counts closely follow the US’s engagement history in the Middle East. The strong deployment association shows the importance of overseas acquisition. The increasing prevalence of CD suggests it is an emerging disease of concern in the US, particularly in the southern states, indicating that further studies on its spread in the US are needed.

**Disclosures:**

All Authors: No reported disclosures

